# Diagnosing problems with imputation models using the Kolmogorov-Smirnov test: a simulation study

**DOI:** 10.1186/1471-2288-13-144

**Published:** 2013-11-20

**Authors:** Cattram D Nguyen, John B Carlin, Katherine J Lee

**Affiliations:** 1 Clinical Epidemiology & Biostatistics Unit, Murdoch Childrens Research Institute, The Royal Children’s Hospital, Flemington Road Parkville, Melbourne, Victoria 3052, Australia; 2 Department of Paediatrics, Faculty of Medicine, Dentistry and Health Sciences, University of Melbourne, Melbourne, Australia; 3 Melbourne School of Population and Global Health, Faculty of Medicine, Dentistry and Health Sciences, University of Melbourne, Melbourne, Australia

**Keywords:** Missing data, Multiple imputation, Model checking, Kolmogorov-Smirnov test, Diagnostics, Simulations

## Abstract

**Background:**

Multiple imputation (MI) is becoming increasingly popular as a strategy for handling missing data, but there is a scarcity of tools for checking the adequacy of imputation models. The Kolmogorov-Smirnov (KS) test has been identified as a potential diagnostic method for assessing whether the distribution of imputed data deviates substantially from that of the observed data. The aim of this study was to evaluate the performance of the KS test as an imputation diagnostic.

**Methods:**

Using simulation, we examined whether the KS test could reliably identify departures from assumptions made in the imputation model. To do this we examined how the p-values from the KS test behaved when skewed and heavy-tailed data were imputed using a normal imputation model. We varied the amount of missing data, the missing data models and the amount of skewness, and evaluated the performance of KS test in diagnosing issues with the imputation models under these different scenarios.

**Results:**

The KS test was able to flag differences between the observations and imputed values; however, these differences did not always correspond to problems with MI inference for the regression parameter of interest. When there was a strong missing at random dependency, the KS p-values were very small, regardless of whether or not the MI estimates were biased; so that the KS test was not able to discriminate between imputed variables that required further investigation, and those that did not. The p-values were also sensitive to sample size and the proportion of missing data, adding to the challenge of interpreting the results from the KS test.

**Conclusions:**

Given our study results, it is difficult to establish guidelines or recommendations for using the KS test as a diagnostic tool for MI. The investigation of other imputation diagnostics and their incorporation into statistical software are important areas for future research.

## Background

Multiple imputation (MI) is becoming increasingly popular as a strategy for analyzing incomplete data [1,2]. MI is a flexible method comprising two main steps: imputation and analysis. During the imputation step, each missing value is replaced with multiple (*m* >1) imputed values drawn from a predictive distribution for the missing values given the observed data. This results in *m* completed datasets, each of which is analyzed separately using standard complete-data methods. The results from the *m* analyses are then combined to give an overall MI estimate. The combination of estimates is most commonly performed using arithmetic rules derived by Rubin [3].

In order to generate the imputed data, the user must specify an imputation model. The imputation model makes use of relationships between the observed values of complete and incomplete variables to generate a predictive distribution from which the imputed data are drawn. The quality of the imputed values rests on how well this imputation model has been formulated. Misspecification of imputation models can lead to biased results [1] and it is important that diagnostic checks are performed to assess the adequacy of imputation models.

Although there have been advances in statistical methods and software for implementing MI, there has been a lag in the development of imputation diagnostics. There are few guidelines in the literature on how users should be checking their imputation models. A few papers have proposed formal model-checking approaches such as posterior predictive checking and cross-validation [4-6]. Informal graphical techniques, such as plotting the imputed and observed variables, have also been recommended [7,8]. However, few of these diagnostics have undergone rigorous evaluation and the majority is not yet available in the standard statistical packages supporting MI.

The Kolmogorov-Smirnov (KS) test has been identified as a potentially useful tool for diagnostic checking in MI [7]. The KS test is a non-parametric procedure for testing whether two samples are from the same population. The test statistic corresponds to the maximum vertical distance between the empirical distribution functions of the two samples [9].

In the context of MI, the KS test has been proposed as a diagnostic tool to compare the distributions of observed and imputed data [7]. For any variable subject to missing data there is an empirical distribution of observed values and after imputation there will be a distribution of imputed values for the cases that had missing values. The proponents of the KS test suggest that variables whose imputed values differ markedly from the observed data be flagged for further investigation [7]. For example, Abayomi et al. [7] applied the KS test diagnostic when performing MI in a dataset consisting of 64 environmental variables from 142 countries. These variables were of interest for constructing an aggregate national measure called the environmental sustainability index (ESI). In total, 19% of the data required imputation and the ESI was estimated based on 10 imputations. To check their imputation models, Abayomi et al. [7] performed KS tests on all imputed variables within a single imputed dataset. They flagged any variables with KS test p-values below 0.05 and examined the flagged variables further using graphical techniques.

An advantage of using the KS test is that it is commonly available in standard statistical software and can easily be applied to imputed data. This test has recently been incorporated into a user-written command in Stata, enabling users to easily compare the distributions of observed and imputed data [10]. Because the KS test is a numerical test, it can also be performed in a semi-automated fashion. The test can be used as a screening device to highlight variables that require in-depth checking [7]. This addresses the challenge of performing manual checks on all variables, particularly when working with large multivariate datasets.

Although the KS test can be used to check imputation models, its performance as a diagnostic tool for MI has not been formally evaluated. For example, it is unclear whether the proposed flagging procedure can successfully identify poorly specified imputation models. Many issues also remain unclear with respect to how KS p-values are to be interpreted and how decisions are to be made based on multiple KS p-values. In particular, it is unclear how KS p-values should be interpreted in light of assumptions about the missing data mechanism [3]. Using the KS test as an imputation diagnostic implies that differences between observed and imputed values are undesirable or problematic. However, there are circumstances where differences are to be expected. When the data are missing completely at random (MCAR), where the probability of data being missing does not depend on the values of the observed or unobserved data, we would expect the distributions of imputed and observed data to be similar and for the null hypothesis of equal distributions to hold. However, when data are missing at random (MAR), i.e. where the probability of missingness is related to the values of the observed variables but not the unobserved variables, we may expect the distribution of the imputations to differ from the observed data. If differences flagged by the KS test are not always of concern, it is difficult to know whether action is required when a flag is raised. Understanding how the KS test behaves under different missing data mechanisms will be important for assessing its usefulness as a diagnostic test.

In the current study, we assessed the performance of the KS test as a diagnostic method for MI. As a motivating example, we applied the KS test when imputing missing data in the Longitudinal Study of Australian Children, a large-scale epidemiological dataset. We then performed a simulation study to formally assess the performance of the KS test as an imputation diagnostic.

### Motivating example

The Longitudinal Study of Australian Children (LSAC) is a national longitudinal study that examines the educational, cognitive, social, mental health, and physical development of Australian children [11]. The specific question of interest within LSAC that we focused on for this investigation was to examine which early childhood (0–3 years of age) risk factors predict conduct problems at age 6–7 years. The outcome of interest was conduct problems at wave 4 (6–7 years) as assessed by the conduct subscale of the Strengths and Difficulties Questionnaire (SDQ), which is a semi-continuous outcome on a scale from 0 to 10 [12]. Potential risk factors were child, family and community factors measured at wave 1 (0–1 year) and wave 2 (2–3 years). This case study was based upon previously published research by Bayer et al. [13]; however, we modified the analysis to distinguish our case study from the published research. The analysis was also simplified as our focus was on the MI diagnostics, rather than the substantive research question.

Linear regression was used to identify early childhood predictors of conduct problems in middle childhood. The following covariates were included in the regression model: mother’s high school completion (yes/no), family socioeconomic position (continuous, range −4.9, 3.0), gender, warm parenting (semi-continuous, range 2.2-5), harsh discipline (semi-continuous, range 1–10), mother’s emotional distress (semi-continuous, range 0–24) and mother’s smoking status (yes/no). For the purpose of this analysis, the sample was restricted to the 4211 cases with observed outcome. The percentage of missing values in the dataset ranged from 0% for gender to 23% for mother’s smoking status and harsh discipline scores (Table 1). Of the 4211 children in the sample, 3175 children (75%) had data available for all covariates.

**Table 1:** Linear regression analysis results for the Longitudinal Study of Australian Children example

**Variable**	**Missing**	**Complete case analysis**		**Model 1**		**Model 2**		**Model 3**		**Model 4**	
	**n (%)**										
		**Coefficient**	**SE**	**Coefficient**	**SE**	**Coefficient**	**SE**	**Coefficient**	**SE**	**Coefficient**	**SE**
Mother completed high school	3 (0.1)	−0.147	0.061	−0.216	0.054	−0.212	0.054	−0.233	0.054	−0.212	0.053
Socioeconomic position	151 (3.6)	−0.192	0.028	−0.183	0.026	−0.184	0.026	−0.184	0.025	−0.180	0.025
Male child	0 (0)	0.236	0.048	0.281	0.043	0.283	0.043	0.284	0.043	0.281	0.043
Warm parenting	251 (6.0)	−0.309	0.059	−0.297	0.053	−0.290	0.055	−0.295	0.054	−0.303	0.054
Harsh discipline score	970 (23.0)	0.252	0.017	0.232	0.018	0.212	0.017	0.247	0.017	0.241	0.018
K6 total score	287 (6.8)	0.033	0.008	0.035	0.007	0.036	0.008	0.032	0.007	0.033	0.008
Smoker	971 (23.1)	0.155	0.069	0.193	0.063	0.199	0.062	0.153	0.077	0.208	0.063

The multiple imputation results were estimated using 20 imputations. The four imputation models were: 1 = “optimal” model, 2 = no outcome variable, 3 = no auxiliary variables, 4 = no de-skewing.

The missing data were imputed using multivariate normal imputation (MVNI), which assumes that all variables in the imputation model jointly follow a multivariate normal distribution [13]. We selected MVNI, because this approach is widely available in standard statistical packages [14]. We return to this decision in the discussion. We imputed the missing data using four different imputation models.

Model 1) This imputation model was considered to be the “optimal” model, as it was constructed based on recommendations in the literature [1,15-17]. We included the outcome variable in the imputation model to prevent associations between predictors and the outcome being biased towards zero [17]. We also included “auxiliary” variables in the model, i.e. variables that are not intrinsically of interest, but can improve the quality of the imputations [15]. The auxiliary variables were the same as those in the analysis model, but measured at different waves. Skewed variables were transformed prior to imputation using a zero-skewness log transform (using the lnskew0 command in Stata 12 [18]) and were back-transformed after imputation. This was based on findings that including skewed variables in an imputation model can lead to bias and poor coverage [16].

Model 2) As model 1, but the outcome variable was omitted.

Model 3) As model 1, but the auxiliary variables were omitted.

Model 4) As model 1, but the skewed variables were not transformed prior to imputation.

For each model, imputed values of continuous and semi-continuous variables were truncated at the minimum and maximum values of the observed data. Adaptive rounding was used to round the imputed values of binary variables [19]. For each imputation model, the missing data were imputed 20 times and the regression analysis was performed on each completed dataset. Rubin’s rules [3] were then used to combine results from the 20 imputed datasets to obtain an overall MI estimate of the regression coefficients.

We used the KS test to check the four imputation models by comparing the distributions of the imputed and observed values of variables that required imputation. KS tests were applied to the continuous and semi-continuous variables only, i.e. family socioeconomic position, warm parenting, harsh discipline and mother’s emotional distress. For each imputation model, separate KS tests were performed on each of the 20 imputed datasets, resulting in 20 p-values for each imputed variable. The 20 p-values were then summarized using medians. All analyses were performed using Stata version 12 [18].

Estimates of the regression coefficients for the complete case analysis and the four multivariate normal imputation models are shown in Table 1. As expected, all four imputation approaches increased the precision of the regression estimates in comparison to complete case analysis. There were differences between the complete case analysis results and the imputation results. For example, the regression coefficient for mother’s high school completion was −0.15 for the complete case analysis, while it was approximately −0.2 for the four imputation models, suggesting that the data were not MCAR. In general, the regression results did not differ substantially across the four imputation models. Differences were more apparent for the variables with large proportions of missingness, such as smoking and harsh discipline (both variables had 23% missing). For example, the coefficient for the smoking variable was 0.15 for model 3, while it was around 0.20 for the other three models.

Table 2 shows the median KS p-values for family socioeconomic position, warm parenting, harsh discipline and mother’s emotional distress for each of the four imputation models. The median KS test p-values were very small for all four imputation models. These results suggest that there were substantial discrepancies between observed and imputed data in all four cases. If using the conventional cut-point of 0.05, as done by Abayomi et al. [7], all variables would be flagged for further investigation.

**Table 2:** Kolmogorov-Smirnov (KS) test p-values for the Longitudinal Study of Australian Children example

**Variable**	**Model 1**	**Model 2**	**Model 3**	**Model 4**
Family socioeconomic position	4.94 × 10^-7^	9.02 × 10^-7^	0.024	2.85 × 10^-6^
Warm parenting	4.81 × 10^-12^	4.81 × 10^-12^	3.38 × 10^-12^	1.73 × 10^-8^
Harsh discipline	2.52 × 10^-5^	6.21 × 10^-5^	8.45 × 10^-6^	6.39 × 10^-15^
Mother’s emotional distress	4.48 × 10^-6^	2.44 × 10^-6^	9.95 × 10^-6^	1.78 × 10^-16^

Results are median KS p-values over 20 imputed datasets. The four imputation models were: 1 = “optimal” model, 2 = no outcome variable, 3 = no auxiliary variables, 4 = no de-skewing.

**Figure 1:**
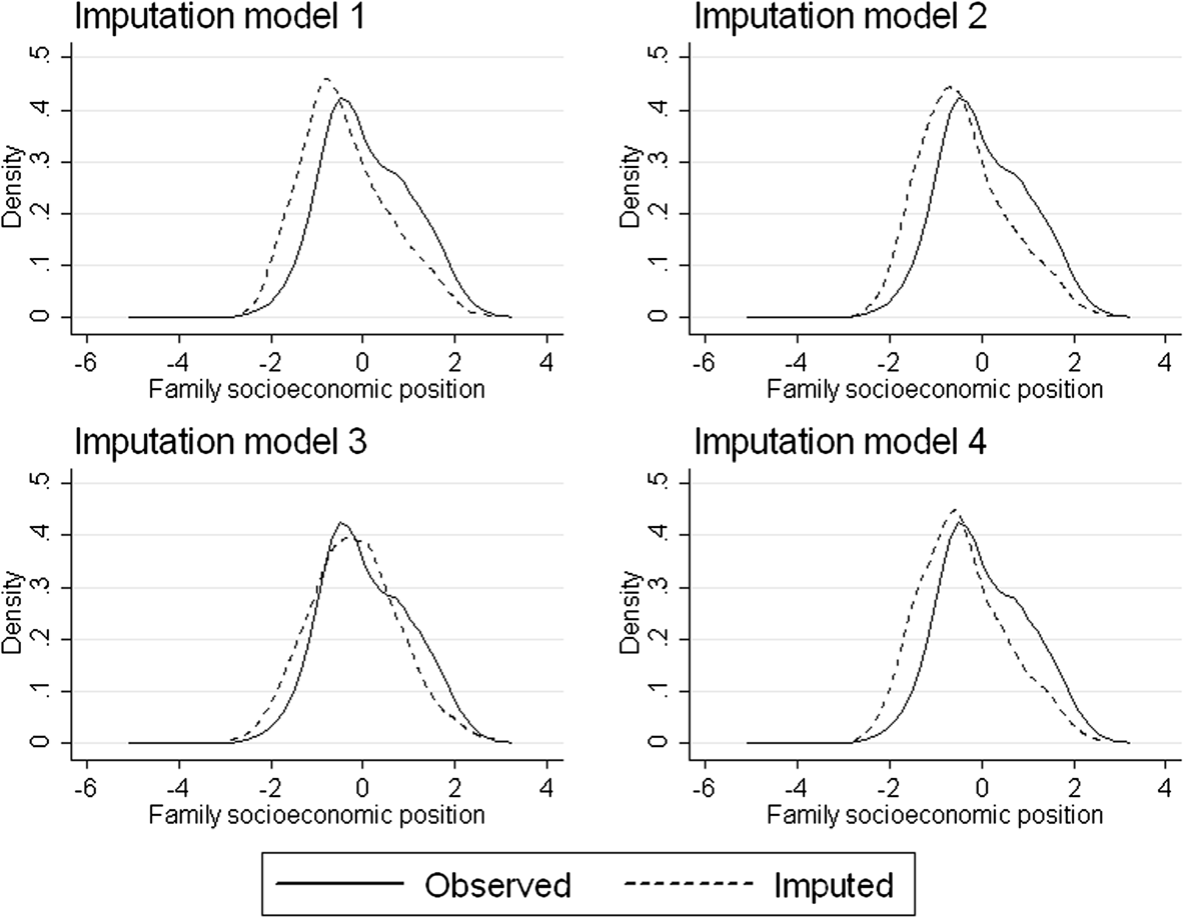
**Results from the Longitudinal Study of Australian Children example.** Density plots of observed (solid line) and imputed (dashed line) data for family socioeconomic advantage. Larger values represent greater socioeconomic advantage. The data from the 20 imputed datasets have been pooled.

One median p-value was notably larger than the other p-values in Table 2. This was for the KS test comparing the observed and imputed values for family socioeconomic status in model 3 (p-value = 0.024). We examined this variable further by graphing density plots of the observed and imputed data from each of the four imputation models, shown in Figure 1. In each of the plots, the observed data are represented by a solid line and the imputed data are represented by a dashed line. For models 1, 2 and 4, the distribution of imputed values was shifted to the left of the distribution of observed values. For model 3, the distributions of observed and imputed data were more similar. This is consistent with the larger magnitude of the median KS test p-value. We would expect model 3 to perform worse than our “optimal” model (model 1), as there was no auxiliary information to impute the missing data. However, the omission of auxiliary variables has resulted in imputed values that are more similar to the observed distribution, leading to a larger p-value. Thus, the KS test diagnostic has not been helpful for detecting models that we would expect to perform worse; it may even be producing smaller p-values for models that we expect to perform better, such as those that include additional variables that improve the plausibility of the MAR assumption [15].

As a result of the LSAC case study, many questions arose in relation to the implementation of the KS test as an imputation diagnostic. In particular, it was unclear whether small KS p-values reflected a model misspecification, or whether identified differences between the observed and imputed data were simply related to the missing data mechanisms. It was also unclear whether there was any relationship between the KS p-values and the “quality” of the MI inferences of primary interest (in our example for the regression coefficients). Motivated by these issues, we performed a simulation study to examine the following research questions, in the context of a simple regression analysis:

1. Do small p-values reliably highlight departures from assumptions made in the imputation model (e.g. multivariate normality)?

2. What is the relationship if any between KS p-values and the bias in the MI estimation of parameters of interest (e.g. regression coefficients)?

## Methods

To address these questions, we performed simulation experiments using a simple univariate regression model consisting of a completely observed outcome variable and an incompletely observed covariate. We examined how the KS p-values behaved under different scenarios of model misspecification, including skewed and heavy tailed data. We also varied the amounts of data missing and missing data mechanisms. For each scenario, the following steps were performed:

1. A covariate *X* and an outcome variable *Y* were simulated.

2. Missingness was introduced into the *X* variable.

3. The missing *X* data were multiply imputed using a normal model (20 imputations).

4. A linear regression of *Y* on *X* was performed on each imputed dataset and the multiple estimates of the regression coefficient were combined into an overall MI estimate.

5. KS tests were performed to compare the distributions of observed and imputed data within each of the 20 imputed datasets and combined to give a median p-value.

6. Steps 1–5 were repeated 1000 times (i.e. 1000 replications).

Details of these steps are outlined below.

### Data generation

For each replication (n = 1000), we generated a single covariate, denoted *X*, from a skew-t distribution with a sample size of 500. Following Azzalini and Capitanio [20], a skew-t random variable is the ratio of a skew-normal variate to the square root of a chi-square variate. A random variable Z is said to be skew-normal if it has the density *f*(z) = 2*ϕ*(z)Φ(αz) where α is the shape parameter and *ϕ* and Φ are the standard normal density and distribution function respectively [20]. As with a standard t-distribution, the skew-t distribution has υ degrees of freedom controlling the weight of the tails. Skewness is controlled by the shape parameter; skewness increases as α increases, and the distribution converges to a half t-distribution as *α* → ∞. When α = 0 the skew-t distribution reduces to the standard symmetric t-distribution.

Three values of α (0, 2, 5) were selected in order to produce data that were not skewed, moderately positively skewed and very positively skewed, respectively. To vary the weight of the tails of the t-distributions, the degrees of freedom (DF) were set to either 3 (i.e. heavy tails) or 1000. Setting DF = 1000 and α = 0 produced data that were normally distributed, which served as a reference scenario for this simulation experiment. Outcomes (*Y*) were generated using the model Y | *X* = *β X + e* where e ~ N (0,1). The regression coefficient *β* was set to 1 which was thus the target (true) value of the parameter of interest.

### Missing data models

Missingness was imposed on the *X* variable at rates of 20%, 50% and 80%. Three missing data models were examined:

1. *Missing completely at random (MCAR):* Missingness was imposed randomly on the *X* variable.

2. *Missing at random (MAR) mild:* The probability of being missing in *X* depended on the value of *Y*, and was determined using the logistic regression specification logit *p*(*X* is missing) = ζ + *ηY*. We set η = 0.2, which corresponded to an increased odds of 22% of being missing with each unit increase in Y. We varied the value of ζ empirically to achieve the *X* proportions of missingness above.

3. *MAR strong:* This was similar to the MAR mild model above, but with a stronger dependency between *Y* and missingness in . Under this model, we set η = 1 in the above specification, which was equivalent to a 2.8-fold increased odds of *X* being missing with each unit increase in *Y*. Again, the value of ζ was varied to achieve missingness rates of 20%, 50% and 80% respectively.

### Multiple imputation and target analysis

For each scenario the missing *X* data were imputed conditional on *Y* using MVNI, which was implemented in Stata release 12 using the *mi impute mvn* command [18]. In this setting, where there is only one incomplete variable, MVNI would be the same as imputation using a univariate linear regression model. We specified the standard default uniform prior for the imputation model parameters and performed 20 imputations for each simulated dataset.

The parameter of interest was the coefficient *β* from the linear regression of *Y* on *X*. The performance of MI in estimating the regression coefficient was assessed using bias and root mean square error [21]. Bias (denoted δ) was calculated as the difference between the average estimate over *n* (= 1000) simulation replications and the true value of the parameter. The root mean square error (RMSE) is a combined measure of bias and variance, which we estimated using $\sqrt{\delta^{2}+\mathrm{SD}{\left(\hat{\beta }\right)}^{2}}$, where δ is our estimate of bias and $\mathrm{SD}\left(\hat{\beta }\right)$ is the empirical standard error of the regression coefficient over the 1000 simulations.

### Kolmogorov-Smirnov test

The KS test diagnostic was used to assess the equality of distributions of the observed and imputed *X* values for each of the scenarios. KS tests were performed separately for each imputed dataset in each replication; so for each replication there were 20 imputed datasets and 20 KS p-values. Because there was only one incompletely observed variable (i.e. *X*), there was only one p-value for each imputed dataset. For each replication within each simulation scenario, we summarized the 20 KS p-values using minima, maxima and medians (where medians of the 20 p-values were used because the distribution of the p-values was skewed for many scenarios). We also examined the proportions of p-values that were below 0.05. The medians of these summaries over 1000 simulations were then obtained.

## Results

**Figure 2:**
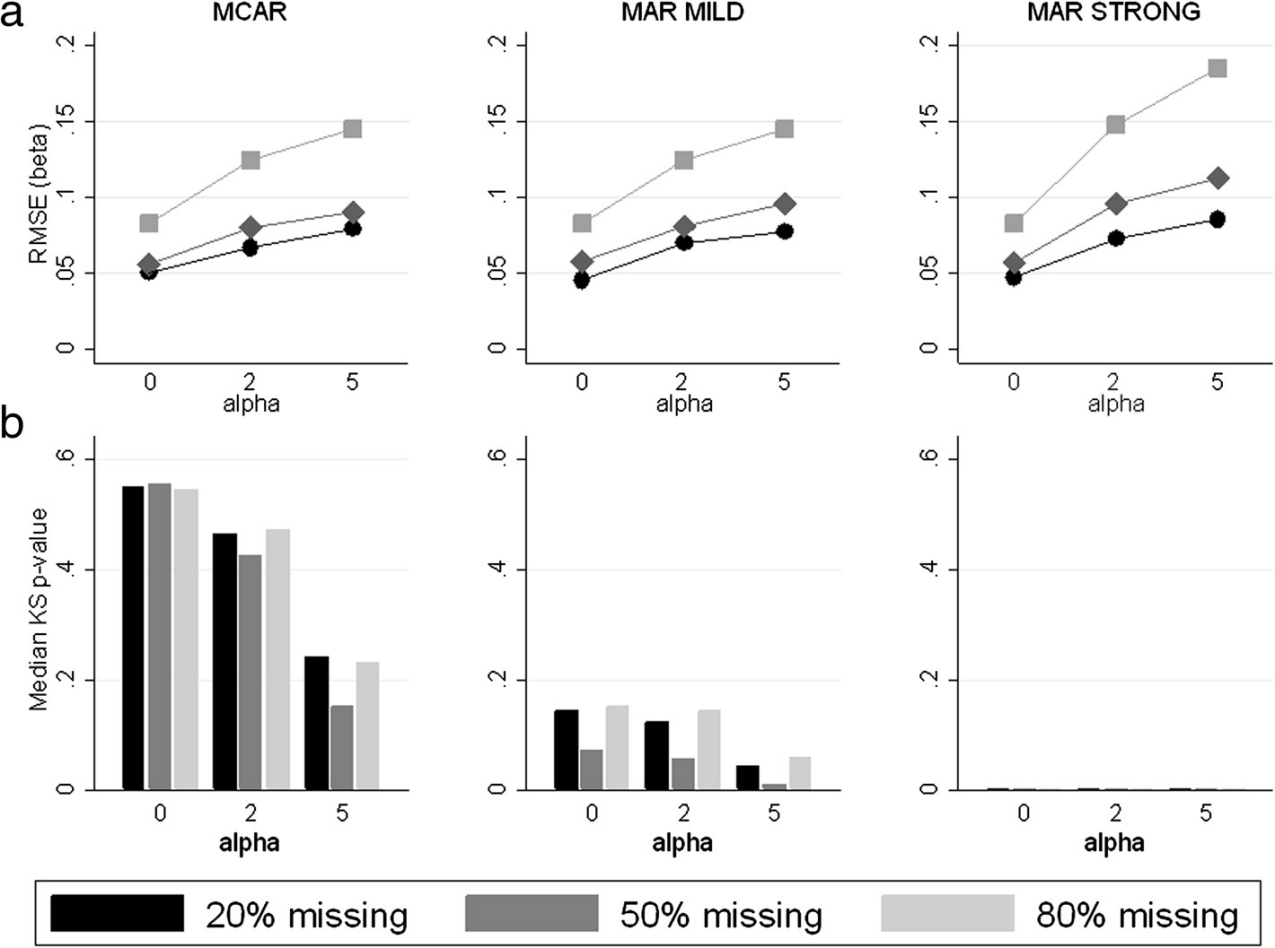
**Simulation study results for degrees of freedom = 1000. a)** Line plots of the root mean square error (RMSE) of the beta coefficient estimates against alpha (the parameter controlling the skewness), and **b)** Bar charts of median Kolmogorov-Smirnov (KS) test p-values across the 20 imputed datasets and 1000 replications against alpha. MCAR = missing completely at random, MAR = missing at random.

Results for the simulation study are shown in Figure 2. The line plots (Figure 2a) are graphs of the mean RMSE of the MI estimate of the regression coefficient across the 1000 simulations against α, which controlled the skewness of *X*. As α increased, the RMSE of the MI estimates also increased. Thus, the performance of MI worsened with increasing departures from multivariate normality. The RMSE also increased with increasing amounts of missing data. This pattern of results was consistent across the three missing data mechanisms and two heavy-tailed scenarios. In Figure 2, we summarize the MI results using RMSE; however, similar findings were seen for bias. Bias was minimal when α = 0 and DF = 1000, and increased with skewness and tail heaviness.

We examined whether the KS test was able to identify when MI was performing badly, i.e. whether the KS p-values became smaller as the RMSE increased. As shown in Figure 2b, when the missing data mechanism was MCAR, the KS test p-values decreased as the RMSE increased. Thus, under MCAR the KS test showed potential value as a diagnostic check. However, when data were MAR, particularly when the mechanism was “MAR strong”, all of the p-values were very small. This was regardless of how skewed the observed data were and whether or not the coefficients had a large RMSE or bias. For example, in the null scenario (DF = 1000, α = 0) when there was 20% missing, all of the median p-values across the 20 imputed datasets for each of the 1000 replicates were below 0.05, even though the bias in the parameter estimate was negligible (−0.0002).

Figure 2b also highlights the sensitivity of KS p-values to the sample size and to the amount of missing data. As seen in the bar charts, the magnitude of the KS p-values was similar for 20% and 80% missing, while it was consistently smaller for 50% missing. Thus, there was a tendency for KS p-values to be smaller when there was around 50% missingness compared to when there were large or small amounts of missing data. This did not reflect the pattern we saw with RMSE, where there was increasing RMSE with increasing amount of missingness.

## Discussion

The current study examined the performance of the KS test as a diagnostic tool for MI using both a case study and a simulation study. The results from the simulation study indicate that the KS test diagnostic was able to detect differences between observed and imputed data when the imputation model was misspecified (as seen when skewed and heavy tailed data were imputed under a multivariate normal model). However, the differences flagged by the KS test diagnostic did not always correspond to problems with MI inference. When there was a strong MAR dependency, the KS test did not specifically identify imputations that led to poor MI estimates, i.e. large RMSE. The KS test flagged all imputation models, even when there was little error in the estimate of the regression coefficient. In this strongly MAR scenario, the KS test did not discriminate between misspecified and more appropriate imputation models.

The simulation results draw attention to the challenge of interpreting the KS p-values in the context of different missing data mechanisms. Differences between imputed and observed values can arise due to model misspecification, but they can also occur when the missingness is not MCAR. It is not possible to disentangle these sources of difference using the KS test p-values.

Using KS p-values to identify potential problems with the imputation model specification in an automated fashion also requires the user to select a significance level at which differences between observations and imputations are to be flagged. This threshold will be context-specific, since the magnitude of the p-values will be influenced by characteristics of the data, and in particular by the sample size. In the environmental dataset analyzed in Abayomi et al. [7], the KS test was applied to a sample of 142 countries. The KS test p-values were below 0.05 in approximately half of the variables examined. In contrast, in our LSAC example we had a sample size of over 4000. With this large dataset, all of our variables would have been flagged if we were to use the conventional significance threshold of 0.05. Abayomi et al. [7] acknowledge that other flagging rules or significance thresholds may be required. As an alternative they suggest examining the 10% of variables whose KS test p-values are the most extreme. This rule would overcome the problem of all variables being flagged but still relies on an arbitrary choice of 10% to be flagged.

In addition to the overall sample size, the magnitude of the p-values can be influenced by the proportion of missing data. Somewhat paradoxically, p-values were consistently smaller when 50% of the data had been imputed, compared to when a larger proportion of the data (80%) had been imputed. This is because the distributional comparison based on a 50:50 split of observed to missing data is more powerful than either an 80:20 or a 20:80 split for the same overall sample size. Thus, not only would users have to consider their sample size when selecting a significance level, but they would also need to take into account the proportion of missing data in each of the incomplete variables. To address this problem, it may be possible to calibrate the p-values to account for the sample size and the proportion of missing data. However, these adjustments to the p-values will not overcome the problem of the dependence on the missing data mechanisms described above.

In the environmental sustainability example, Abayomi et al. [7] performed the KS test on a single imputed dataset. However, there is little guidance on how this test should be carried out on multiply imputed data. One possibility is to pool the imputed data and perform a single KS test for each imputed variable. This approach will inflate the sample size, introducing an arbitrary dependence on the number of imputed datasets. Alternatively, the KS tests can be performed on each imputed dataset as used in the current study, i.e. if there are m imputed datasets, then m KS tests can be performed, resulting in m KS p-values per imputed variable. This raises questions about how judgments regarding model adequacy should be made based on these multiple p-values. In both the LSAC application and the simulation study, we performed 20 imputations and summarized the multiple p-values using medians (using medians due to the highly skewed nature of the p-values). However, there are a number of approaches that we could have used to judge the imputation models based on the 20 p-values. For example, we could have made decisions based on a single extreme value, or we could have considered the proportion of the 20 p-values that were below a specified threshold. There is also the question of whether adjustments for multiple testing are required when performing the KS test on multiple variables with incomplete data. These are further decisions that users would have to make if they applied the KS test diagnostic to imputed data. However, given the shortcomings of the KS test described above, we would investigate other model checking approaches, rather than develop the KS test diagnostic further.

The KS test diagnostic focuses on distributional differences between the observed and imputed data. In the MI literature, there are other proposed diagnostics that target differences between the observed and imputed data in more specific characteristics, such as the location and spread. For example, Stuart et al. [22] propose flagging imputation models if i) the absolute difference in means between the observed and imputed data is greater than 2 standard deviations, or ii) the ratio of the variances of the observed and imputed values is less than 0.5 or greater than 2. Similarly, classical tests of differences in variances or means (e.g. F-test, t-test and non-parametric counterparts) could be used. Although we did not assess these tests in our study, we believe that our general conclusions will still apply. Under MAR, we do not expect the imputed data to resemble the observed data. In fact, we may be relying on MI to recover these differences based on information in the observed data. It may be useful to explore how the observed and imputed data differ (e.g. through plotting or tabulating summary statistics). However, we do not recommend automatic flagging of differences using numerical tests, because the discrepancies between observed and imputed data do not necessarily signal a problem.

A limitation of the simulation study presented here is that it does not represent a realistic scenario, since our datasets consisted only of a single covariate and an outcome variable. However, we decided to use this model to examine the performance of the KS test diagnostic within a simple setting. Given the intrinsic problems with the KS test in this simple scenario, the results of the test would only be harder to interpret in a more realistic situation where there would be additional complications, as well as issues with multiple testing when using the KS test on a number of incomplete variables. We also decided to implement MI using MVNI, as this is a widely used approach that is available in standard statistical software. In this simple context, similar results would be expected from chained equations [23,24], another popular and widely available approach to multiple imputation, as these methods are equivalent when there is missingness in a single continuous variable. In this paper we did not compare these methods, as the focus was on the value of the KS test as an imputation diagnostic.

A final limitation of the simulation study is that method used to generate the data means that the imputation models were technically misspecified in all scenarios. In the simulations, we first generated *X* and then obtained *Y* using a linear regression of *Y* on *X*. This was consistent with our analysis model, which was also a linear regression of *Y* on *X*. This meant that we were able to use the true value of the regression parameter as a reference for evaluating our imputation models. We then induced missingness in the covariate *X*, and imputed the missing values using the imputation model *X* | *Y*. We selected this approach, because MI tends to be utilized for imputing covariates, rather than outcomes in epidemiological analyses and we therefore believed that this scenario had greater generalizability. However, the fact that the imputation model is not the same the data-generating model, may have contributed to bias in the MI results for all of the scenarios considered. Despite this, we are confident that the incompatibility of the data generation and imputation models does not affect our assessment of the KS test as an imputation diagnostic. Our focus in this study was to examine whether the KS test was able to identify the models that produced the largest bias and RMSE. The problems we identified with the KS test were not linked with the data generating process.

## Conclusions

Our simulation study demonstrates the challenges of summarizing and interpreting KS test p-values. In particular, it is not possible to determine whether extreme p-values have arisen due to model misspecification or because data were MAR. Given that MI is favored as a missing data technique when data are MAR, the value of the KS test as an MI diagnostic is questionable. The magnitude of the KS p-value was also influenced by the sample size and proportion of data missing. Given these considerations, it is difficult to establish guidelines that would enable us to recommend applying the KS test as an MI diagnostic.

Further development of other model-checking techniques would be valuable. In particular, the evaluation of diagnostics such posterior predictive checking [4,5] and graphical methods [7,25] is an important area of research. It is also vital that these diagnostics are incorporated into statistical software to improve the accessibility of these techniques and to encourage the practice of model-checking when performing MI.

## Abbreviations

DF: Degrees of freedom; ESI: Environmental sustainability index; KS test: Kolmogorov-Smirnov test; LSAC: Longitudinal study of Australian children; MAR: Missing at random; MCAR: Missing completely at random; MI: Multiple imputation; MVNI: Multivariate normal imputation; RMSE: Root mean square error; SDQ: Strengths and difficulties questionnaire; SE: Standard error.

## Competing interests

The authors declare that they have no competing interests.

## Authors’ contributions

All authors participated in the design of the simulation study and the interpretation of the results. CN performed the case study analysis and conducted the simulations. CN wrote the first draft of the manuscript and prepared all tables and graphs. All authors read and contributed to the final manuscript.

## Pre-publication history

The pre-publication history for this paper can be accessed here:

http://www.biomedcentral.com/1471-2288/13/144/prepub
